# Production and characterization of Newcastle disease antibody as a reagent to develop a rapid immunodiagnostic test tool

**DOI:** 10.14202/vetworld.2018.895-901

**Published:** 2018-07-05

**Authors:** Dwi Desmiyeni Putri, Ekowati Handharyani, Retno Damajanti Soejoedono, Agus Setiyono, Okti Nadia Poetri

**Affiliations:** 1 Study Program of Animal Husbandry, Department of Animal Husbandry, State Polytechnic of Lampung, Indonesia; 2 Department of Veterinary Clinic Reproduction and Pathology, Faculty of Veterinary Medicine, Bogor Agricultural University, Indonesia; 3 Department of Animal Diseases and Veterinary Public Health, Faculty of Veterinary Medicine, Bogor Agricultural University, Indonesia

**Keywords:** antibody, cross reaction, reagent, SDS-PAGE

## Abstract

**Aim:**

This research was conducted to produce and characterize ND antibody as reagent candidate to develop a rapid immunodiagnostic test tool.

**Materials and Methods:**

Four New Zealand White rabbits were used in this study and divided into two groups. First group was injected by Sato ND antigen, and second group was injected by genotype VII ND antigen. This study is divided into three steps: (a) ND antibody production, (b) ND antibody purification, and (c) ND antibody characterization. First group was rabbit injected by Sato NDV (5×10^8.25^ egg lethal doses (ELD)_50_/ml) and second group was injected by genotype VII NDV (5×10^6.5^ ELD_50_/ml). Antigen induction was performed by subcutaneous administrated for first (day 1) and second (day 14) injection and intravenous administrated for third (day 30) injection. Blood was collected on day 8 after third injection.

**Results:**

Antibody production increased on second antigen injection and reached a peak on day 9 after second antigen injection. Sato and genotype VII ND antibody can be produced without adjuvant within 38 days with the highest titer 2^10^. Based on antibody titer data, both antigens induced antibody production in a similar trend. The characterization antibody by SDS-PAGE indicated that molecular weight of immunoglobulin G (IgG) is 154.93 kDa (whole IgG), heavy chain 54.39 kDa, and light chain 27.74 kDa. ND antibodies have specificity to homologous and heterologous NDVs in varying virulence.

**Conclusion:**

Sato and genotype VII ND antibodies have been successfully produced within 38 days without adjuvant. Specificity of ND antibodies to NDVs in varying virulence and cross-reaction between Sato ND antibody and genotype VII ND antibody indicates that the characterized ND antibodies can be used as a reagent to develop rapid immunodiagnostic test tools.

## Introduction

Newcastle disease (ND) is one of the important poultry diseases in the world which caused by ND virus (NDV) and also known as avian paramyxovirus type-1 (APMV-1) [1,2]. The virus has six major proteins: nucleocapsid protein (N), phosphoprotein (P), matrix protein (M), fusion protein (F), hemagglutinin-neuraminidase protein, and large polymerase protein (L) [3]. Based on genotype, NDV can be divided into two classes. Class 1 is commonly found in waterfowl and avirulent in chicken, whereas Class 2 NDV consists of 16 genotypes which are commonly found in chicken, pet birds, and wild poultry [4].

The first ND outbreak was occurred in Java Island (Indonesia) and the United Kingdom, reported in the mid-1920s [5]. In a few years, the disease spread worldwide and became endemic in many countries [6]. Currently, almost all areas in Indonesia are infected, and until now, there is no free area ND in Indonesia.

In developing countries, the losses caused by ND outbreaks are not only due to high mortality but also additional expenditure used for prevention and control programs, i.e., vaccination, biosecurity, and depopulation [7]. However, the right control strategy can be done if the disease diagnosis can be done quickly and precisely.

Disease diagnosis can be done through a series of activities involving observation of clinical symptoms, histopathological lesion, and laboratory tests [8,9]. However, the similarity of clinical symptoms and gross lesions in ND-infected chicken with other diseases can be confusing in determining the proper diagnosis. Recently, we have developed a method to detect NDV using reverse transcriptase-polymerase chain reaction (RT-PCR) diagnostic tool [10]. However, RT-PCR method requires special facilities and high cost relatively that is one of the inhibiting factors in decision-making to control ND outbreak in the field. Therefore, in field, fast and affordable diagnostic test tool is necessary.

This research was conducted to produce and characterize ND antibody as reagent candidate to develop rapid immunodiagnostic test tool.

## Materials and Methods

### Ethical approval

This research has been approved by the Animal Care and Use Committee of Research and Community Services Institution, Bogor Agricultural University, with approval number: 3-2016 RSHP FKH-IPB.

### NDV

Two NDVs were used in this study. First virus was NDV/Ck/BGR/2011 obtained from repository of the Laboratory of Immunology, Faculty of Veterinary Medicine, Bogor Agricultural University, which categorized as virulent NDV and belongs to genotype VII NDV [11,12]. Another virus was Sato NDV which obtained from the National Veterinary Drug and Assay Laboratory, Gunung Sindur, Bogor, Indonesia.

### Experimental animal

Experimental animals used in this study were four New Zealand White rabbits aged 10-16 weeks with an average body weight of 2.5 kg obtained from the Indonesian Animal Husbandry Research Institute, Ciawi, Bogor, Indonesia.

### Research design

This study is divided into three stages: (a) ND antibody production, (b) ND antibody purification, and (c) ND antibody characterization.

#### ND antibody production

ND antibody production was performed in four New Zealand White rabbits which divided into two groups. First group was rabbit injected by Sato NDV (5×10^8.25^ embryo lethal dose [ELD]_50_/ml) and second group was injected by genotype VII NDV (5×10^6.5^ ELD_50_/ml). Antigen was administrated by subcutaneous in first (day 1) and second (day 14) injection and intravenous in third (day 30) injection. Blood was collected on day 8 after third injection. Bleeding is performed by taking blood intracardially after the rabbits had been anesthetized with a ketamine (35 mg/kg BB) and xylazine (5 mg/kg BB) mixture. The collected blood was prepared as follows: The blood samples were stored at room temperature (± 25°C) for an hour and continued storage at 4°C overnight. The obtained serum was separated manually by aspiration and was completed by centrifugation at 2500 rpm for 15 min. The obtained serum was stored in 1.5 ml polypropylene microcentrifuge tubes and stored at −20°C until use. To analyze antibody titer by hemagglutination inhibition test, blood was collected on day 12 after the first injection, days 5, 9, and 16 after second injection, and days 3, 5, and 8 after third injection.

#### ND antibody purification

The purification of ND antibody was performed by two steps. The first step is precipitation with ammonium sulfate according to Duong-Ly and Gabelli [13]. Precipitation using ammonium sulfate (4.1 M) was carried out by adding ammonium sulfate solution to serum and then incubating overnight at 4°C. The precipitation result was centrifuged at 3.000× *g* for 20 min. The obtained filtrate or pellet was reconstituted by phosphate buffered saline (PBS) pH 7.4 to obtain one-fourth of antibody volume. Subsequently, dialysis was performed by preparing a precipitated serum in a dialysis sac (Spectra/Por, USA) and stirred in pH 7.4 of PBS for 24 h at 4°C and each 8 h PBS solution was replaced. Following the first step, the next antibody purification process used protein A purification kit (Sigma, USA) according to the manufacturer’s instructions. Purification results were examined by sodium dodecyl sulfate-polyacrylamide gel electrophoresis (SDS-PAGE) method. The protein concentration was calculated using coefficient 1.36 at 280 nm wavelength [14].

#### ND antibody characterization

Purified ND antibody molecular weight was determined by SDS-PAGE method [15] using 12% polyacrylamide concentration of separating gel and 4% concentration of stacking gel [16]. The antibody sample (5 μl) was added to 5 μl sample buffer (containing Tris/SDS, bromophenol blue, DTT, and glycerol) and then heated at 60°C for 5 min. A total of 10 μl antibody samples and 5 μl protein markers (PageRuler Prestructive Protein Ladder, Thermo Scientific, USA) were filled in each well. Protein separation was performed by electrophoresis at 100 V for 180 min. The electrophoresis gel was stained with Coomassie Blue for 30 min followed by destaining for 24 h.

Agar gel precipitation test (AGPT) was used to determine the specificity of ND antibody. Specificity of ND antibody was evaluated to some characterized ND isolates [11] and other antigens such as avian influenza (AI), infectious bronchitis (IB), and infectious bursal disease (IBD). The antigen and antibody reaction was showed as precipitation line in agarose gel.

## Results

### ND antibody production

**Figure-1:**
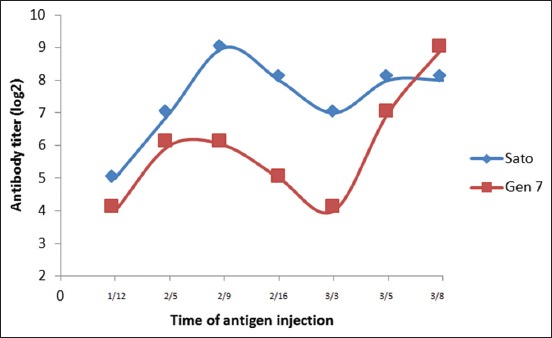
Titer of Newcastle disease antibodies after antigen injection.

The ND antigens which used in this study were Sato NDV and genotype VII NDV [11]. ND antigens injection were performed 3 times, and serum was collected on day 8 after third injection. Antibody titer result after ND antigen injection in each group was determined by HI test (Figure-1). Based on HI titer, rabbits injected with Sato ND antigen and genotype VII ND antigen showed that antibodies were detected on day 12 after first injection, although the antibody titer in each group is still low 2^4^-2^5^. The antibody titer increased after second antigen injection (first boosting) and reached a peak on day 9 in group which injected with Sato ND antigen and on day 5 in rabbits which injected by genotype VII ND antigen. After the third antigen injection, antibody production in rabbits which injected by genotype VII ND increased on day 3 and continued until day 8; nevertheless, antibody production in the rabbits which injected by Sato ND antigen is still constant after day 5.

### ND antibody purification

In this study, the antibody purification was done in two steps using ammonium sulfate (4.1 M) and protein A purification kit (Sigma). The antibody concentration on rabbit serum was determined by UV/Vis spectrophotometer at 280 nm wavelength [17,18]. Utilization of ammonium sulfate 40% is expected to precipitate immunoglobulin G (IgG) optimally. Based on the UV/Vis spectrophotometer, the result showed the Sato ND antibody concentration of 1.76 µg/µland genotype VII ND antibody concentration of 1.82 µg/µl.

**Figure-2:**
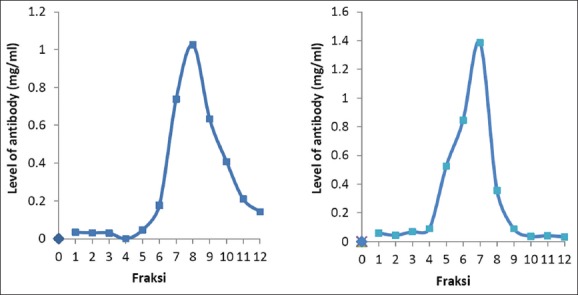
Pattern of Newcastle disease (ND) antibody fraction after purification; (a) Sato ND antibody; (b) Genotype VII ND antibody.

The antibody purification using protein A purification kit produced several fractions. The fraction pattern of protein A purification result was different in each antibody. Sato ND antibody has the higher antibody concentration in fraction 7, 8, and 9 (Figure-2a), and the total amount of purified Sato ND antibody was 3.476 µg/µl (Table-1). The higher concentration of ND genotype VII antibody found in fractions 5, 6, and 7 (Figure-2b), and the total amount of purified ND antibody concentration was 3.574 µg/µl (Table-1).

**Table-1:** Absorbance rate and antibody level after purification.

Fraction	Sato ND antibody		Genotype VII ND antibody	
	
	Absorbance value (280 nm)	Levels of antibodies (mg/ml)	Absorbance value (280 nm)	Levels of antibodies (mg/ml)
1	0.046	0.034	0.079	0.058
2	0.042	0.031	0.060	0.044
3	0.041	0.030	0.094	0.069
4	0.074	0,054	0.122	0.089
5	0.064	0.047	0.714	0.525
6	0.241	0.177	1.150	0.846
7	1.003	0.738	1.885	1.386
8	1.394	1.025	0.486	0.357
9	0.862	0.634	0.122	0.089
10	0.555	0.408	0.055	0.041
11	0.285	0.210	0.050	0.037
12	0.193	0.142	0.045	0.033
	Total	3.476		3.574

### ND antibody characterization

**Figure-3:**
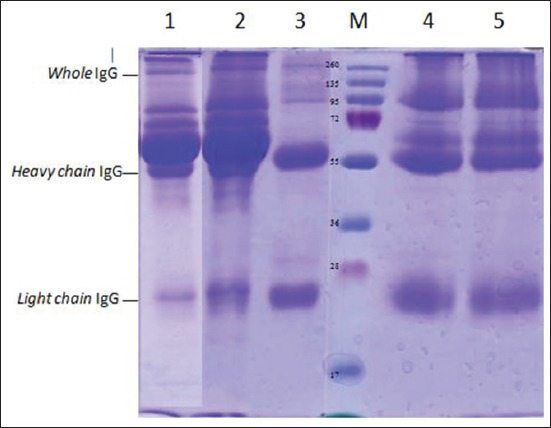
Profile of sodium dodecyl sulfate-polyacrylamide gel electrophoresis Newcastle disease (ND) antibody before purification (1-2) and after purification with protein purification kit A (4-5). Protein marker (M); Sato ND antibody (1 and 4); Genotype ND VII antibody (2 and 5); and commercial standard antibody (3).

A purified antibody with ammonium sulfate (4.1 M) and protein A purification kit was analyzed to determine molecular weight by SDS-PAGE. Electrophoresis results of SDS-PAGE showed that antibody purification with ammonium sulfate had 7 protein bands and serum which had passed through 2 purification steps had 4 protein bands only, while standard antibody had three protein bands (Figure-3). Determination of molecular weight in SDS-PAGE bands was performed by making a linear curve on the calculation of relative mobility value (Rf) and protein molecular weight molecule logarithm. Relative mobility (Rf) calculation and band molecular weight logarithm were obtained through linear regression curve with equation y=−0.2165x + 2.2919; R^2^=0.9141 (Table-2). Based on the regression equation, we found that the molecular weight of standard antibody was 154.93 kDa, 54.39 kDa, and 24.74 kDa for whole IgG, heavy chain IgG, and light chain IgG, respectively (Table-3).

**Table-2:** Determination of equations based on linear curves.

Rf (cm)	BM (kDa)	Log BM
0.31	260	2.4150
0.65	135	2.1303
1.11	95	1.9777
1.64	72	1.8573
2.23	52	1.7160
2.96	42	1.6232
3.58	34	1.5315
4.47	26	1.4150

y=−0.2165x+2.2919

**Table-3:** Calculation of protein molecular weight band in SDS-PAGE analysis of ND antibody.

Rf (cm)	BM (kDa)	Log BM
Ammonium sulfate		
0.47	154.93	2.19
1.11	112.61	2.05
1.36	99.42	2.00
1.91	75.58	1.88
2.33	61.30	1.79
2.57	54.39	1.74
4.15	24.74	1.39
Protein A		
0.47	154.93	2.19
2.33	61.30	1.79
2.57	54.39	1.74
4.15	24.74	1.39

SDS: Sodium dodecyl sulfate-polyacrylamide gel electrophoresis, ND: Newcastle disease

### Specificity test of antibodies

**Figure-4:**
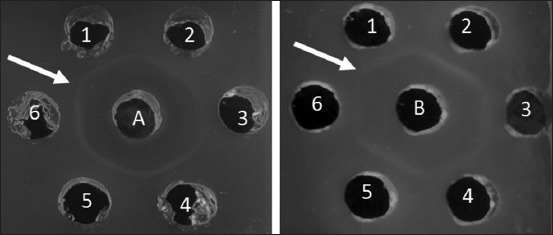
Antibody specificity test with agar gel precipitation test; A. Sato ND serum; B. Genotype VII NDserum; 1-6 Antigen ND. (1) ND/Ck/LG/15; (2) ND/Ck/GS/14; (3) ND/Ck/JP/14; (4) ND/Ck/CJR/15; (5) ND/Ck/Bogor/15; (6) ND/Ck/TRG/15. Arrow (): Precipitation line.

**Figure-5:**
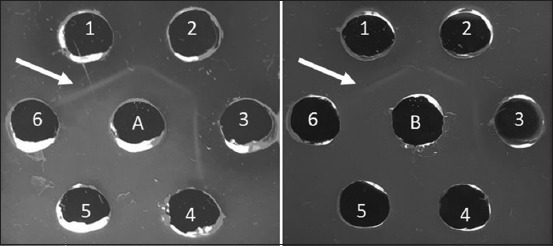
Antibody specificity test with agar gel precipitation test; (a) Sato ND serum; (b) Genotype VII ND serum; (1) Sato ND; (2) Genotype VII ND; (3) Lasota ND; (4) avian influenza virus; (5) infectious bronchitis virus; (6) infectious bursal disease virus. Arrow (): Precipitation line.

Sato and genotype VII ND antibodies were evaluated for specificity to some characterized ND isolates [11] and other antigens such as AI, IB, and IBD using AGPT. The indicator of antigen-antibody reaction at this stage was precipitation line in agarose gel. Based on AGPT result, the precipitation line was formed on all homologous and heterologous ND antigens with varying degrees of virulence (Figure-4), whereas in wells given AI, IB, and IBD antigens, we cannot found the precipitation line (Figure-5).

## Discussion

Recently, the production of antibody in laboratory animals has become an essential part of many research projects. Investigators preparing to produce antibodies are confronted with a number of complex choices, some of which may be critical for success. The main goal in antibody production is to obtain high-titer, high-specificity antibody and still concerned in animal welfare. An antibody which produced should be induced by characterized antigen and also be characterized before used as a reagent in the immunodiagnostic test.

In this study, antibody has been successfully produced within 38 days with 3 times antigen injection. First injection in antibody production aims to introduce antigen to B cell; second and third injections are booster to increase antibody production by B cells [19,20]. In general, antibody begins to be detected in serum on day 6-7 after antigen exposure [21]. In the first antigen injection, the rabbits which injected by Sato ND antigen produced higher antibody than rabbits which injected by genotype VII ND antigen. The number of viruses were injected caused this phenomenon, the rabbits which injected by Sato ND antigen received 5×10^8.25^ virus/ml ELD_50_, while the rabbits which injected by genotype VII ND antigen received 5×10^6.5^virus/ml ELD_50_ only. The third injection was performed after ND antibody titer decreased on day 16 after second injection. The second antigen exposure (booster) will activate memory cells and produce antibody faster as a secondary response [22]. In general, reinjection of antigen (booster) is performed at weeks 3 and 6 after first injection and when antibody titers begin to decline [23]. Based on Figure-1 showed that, antibody titer of rabbits which injected by Sato ND antigen was still high (27) when third antigen injection. Antigens that given will be neutralized by antibody, so only a few antigens can induce subsequent immunologic responses. Neutralization is antibody activities to bind and neutralize the viral surface (virion) to decrease virus ability to infect [24].

Antibody production in both the groups increased rapidly after the third injection by intravenous administrated route. The effectivity of the immune response to produce antibody is determined by the speed of antigen reaching lymphoid organ. By intravenous immunization, antigens reach to spleen and lymph nodes immediately [25]. Antibody production in vaccinated animals may be affected by several factors including immunogenicity, antigen quality and quantity, antigen form and solubility, animal species, and immunization route[26]. ND antibody production in this study was done in 38 days only and more economical with no adjuvant needed. Samiullah *et al*. [27] showed that antibody to APMV-1 can be produced within 91 days using adjuvant and reach titer 1024 (2^10^) with 4 and 5 times injection.

The antibody has to be separated from another component in serum before be characterized. Separation of antibodies from complex mixtures can be achieved using some purification method [28]. In this study, antibody passed two-step purification. First step of antibody purification used ammonium sulfate. Antibody purification using ammonium sulfate is one of the oldest, easiest, and economical methods for purifying antibodies [29]. Ammonium sulfate has a higher solubility than other phosphate salts, which become preferred solutions for precipitation. The principle of antibody purification by ammonium sulfate is ammonium sulfate ability to bind IgG [30]. According to Ausubel *et al*. [15], saturation 35-45% of ammonium sulfate can precipitate 80-90% of IgG in rabbit and sheep serum.

The second step of antibody purification was using protein A purification kit. Protein A is a surface protein in *Staphylococcus aureus* [31] which has 5 domains E, D, A, B, and C base on N-terminus. Each domain of protein A is able to bind Fc fragment of IgG [32]. Purified antibodies were characterized by SDS-PAGE to determine the molecular weight of antibody. According to Johnson [33], molecular weight of IgG ranges from 150 to 160 kDa. Immunoglobulin G has a molecular weight of monomer structure 146,000 daltons, and it is the main antibody of the secondary immune response [34]. Chemical treated IgG molecules, such as SDS, will break the disulfide bond and cause the IgG molecules to break down into four separate polypeptide chains. These chains are “heavy” chain which has a molecular weight of about 50 kDa and two other “light” chains which have a molecular weight of about 25 kDa [35,36]. In serum which purified by ammonium sulfate only, are still found some substances like transferrin, albumin, and other proteins at molecular weight 112.61 kDa, 99.42 kDa, 75.5 kDa, and 61.30 kDa. Transferrin is a protein found in serum with a molecular weight of 75 kDa, and albumin has a molecular weight of 60 kDa [37].

Proteins revealed by SDS-PAGE were not specific yet; therefore, specificity test is required to obtain a specific protein. In this study, antibody specificity was evaluated by AGPT. An antibody which induced by injection whole or partial antigen called as a polyclonal antibody. Polyclonal antibodies are antibodies that have a complex mixture of antibodies with different specificity, affinity, and isotype. Polyclonal antibodies have the function for binding various epitopes on the surface of the inducing antigen molecule [38]. Polyclonal antibodies react with different epitopes (antigen determinants) in multireactivity leading to cross-reactions. Cross-reaction occurs if the same epitope from different antigens which structurally similar are identified by antibodies. This principle can also be used to determine the relationship between antigen molecules [35]. NDV (APMV-1) is one serotype [7], so it will have a cross-reaction with the heterologous NDV [27]. Based on AGPT, the result showed that ND antibody which produced in this study could be used as a reagent to developed rapid immunodiagnostic test tool for testing either homologous ND or heterologous NDVs. Furthermore, these ND antibodies which produce in short time without adjuvant can be used commercially in developed immunodiagnostic test tools for ND. These future diagnostic kits can provide a fast, easy, cheap, and accurate detection of NDVs. Finally, the produced antibodies can be sold commercially as a positive control for immunological tests in laboratories.

## Conclusion

The study succeeded in producing ND antibodies from Sato and genotype VII ND antigens. ND antibodies can be produced within 38 days without adjuvant with the highest titer of 2^10^. The characterization antibody by SDS-PAGE indicated that molecular weight of IgG is a 154.93 kDa, with heavy chain 54.39 kDa and light chain 27.74 kDa. These antibodies are specific to homologous and heterologous NDVs with varying degrees of virulence.

## Authors’ Contributions

DDP executed the work (collection of data, analysis, and writing of manuscript); EH participated in conception and designed the study and drafting of the manuscript; AS participated in designing the study and drafting of the manuscript; RDS participated in designing the study, analysis of data, and drafting of the manuscript, ONP participated in analysis and interpretation of data and writing of manuscript. All authors read and approved the final manuscript.
